# Translation of the Pasieka’s Parathyroid Assessment of Symptoms Questionnaire (PAS-Q) for Use in the Greek Population

**DOI:** 10.3389/fendo.2022.758885

**Published:** 2022-03-04

**Authors:** Georgios Tzikos, Christina Manani, Fotini Adamidou, Alexandra Menni, Moysis Moysidis, Despoina Tsalkatidou, Angeliki Chorti, Kalliopi Kotsa, Konstantinos Toulis, Panagiotis Anagnostis, Antonios Michalopoulos, Theodosios Papavramidis

**Affiliations:** ^1^ ^1st^Propedeutic Department of Surgery, AHEPA University Hospital, Aristotle University of Thessaloniki, Thessaloniki, Greece; ^2^ Department of Endocrinology, Diabetes and Metabolism, Hippokration Hospital of Thessaloniki, Thessaloniki, Greece; ^3^ ^1st^Department of Internal Pathology, AHEPA University Hospital, Aristotle University of Thessaloniki, Thessaloniki, Greece; ^4^ Department of Endocrinology, 424 Military Hospital, Thessaloniki, Greece; ^5^ Unit of Reproductive Endocrinology, ^1st^ Department of Obstetrics and Gynecology, Medical School, Aristotle University of Thessaloniki, Thessaloniki, Greece

**Keywords:** primary hyperparathyroidism, quality of life, Pasieka’s Questionnaire, translation, adaptation

## Abstract

**Introduction:**

In Europe, primary hyperparathyroidism is mainly considered an asymptomatic disorder, although there is evidence that patients’ health-related quality of life is impaired. This aspect is mostly evaluated using Pasieka’s Questionnaire: a disease-specific diagnostic tool. The purpose of this study was to translate the Pasieka’s Questionnaire into the Greek language and adapt it to the Greek population.

**Materials and Methods:**

Pasieka’s Questionnaire consists of 13 questions. Two bilingual, native Greek experts were selected for step one, each of whom offered a blinded Greek version of the questionnaire. In the second step, these two versions were merged into one which was retranslated back into the English language (step three) by two bilingual translators (English native speakers). In the fourth step, a committee was formed to draft the pre-final version of the questionnaire which was then submitted to the co-authors for final approval. Finally, after the approval of the final version, 50 patients with primary hyperparathyroidism were recruited for the pilot study of the questionnaire.

**Results:**

All 13 questions of the Pasieka’s Questionnaire were translated without any major discrepancy. A high level of internal consistency was achieved (Cronbach’s alpha was 0.904) and agreement between test-retest was excellent for every question.

**Conclusion:**

The Greek version of Pasieka’s Questionnaire was validated and can be applied to evaluate the health-related quality of life of patients with primary hyperparathyroidism in Greek-speaking populations.

## 1 Introduction

Primary hyperparathyroidism (PHPT) is a common endocrine disease, characterized by a generalized imbalance of calcium and phosphate, due to the excessive secretion of the parathyroid hormone (PTH), commonly caused by a solitary benign adenoma, and less frequently by either multiple adenomas or hyperplasia, and rarely by parathyroid carcinoma. Its prevalence and clinical presentation vary from country to country. In Europe, PHPT occurs primarily as an asymptomatic disorder. Its classical presentation involves the skeleton and kidneys, while non-classical manifestations include cardiovascular, neurocognitive, gastrointestinal, and psychiatric disorders. Any or all of these manifestations can impact and impair quality of life (QoL) (1).

Pasieka’s parathyroid assessment of symptoms Questionnaire (PAS-Q) is a self-administered questionnaire, which was developed in Canada specifically to assess the symptoms of PHPT, reflecting patients’ QoL. It is considered a useful method to evaluate PHPT patients’ QoL by assessing the degree to which disease-specific symptoms affect their lives (2). PAS-Q was originally designed by Pasieka et al. and validated by its application among Canadians with PHPT.

The purpose of this study was to translate the PAS-Q into the Greek language and adapt its use to Greek patients with PHPT.

## 2 Material and Methods

### 2.1 The PAS-Q

The PAS-Q consists of a set of 13 questions (2). For each question a patient puts a mark on a line representing a sliding scale of 0-100 relating to the severity of the symptoms they experience (0 being symptom-free and 100 being maximum severity). The line is 100mm in length and the position of the individual’s mark is measured in mm from the 0 end of the line, thus giving a score between 0-100 for each question. The total PAS-Q score is equal to the sum of all the individual scores from each question, with a maximum of 1300 and a minimum of 0. The higher the total score, the more impaired the patient’s QoL is considered to be. The 13 questions (Q) evaluate the frequency and degree of the following items: Q1: bone pain, Q2: tiredness, Q3: mood swings, Q4: feeling ‘‘blue’’ or depressed, Q5: abdominal pain, Q6: feeling weak, Q7: feeling irritable, Q8: pain in the joints, Q9: being forgetful, Q10: difficulty getting out of a chair or car, Q11: headaches, Q12: itchy skin and Q13: being thirsty.

### 2.2 Translation of the PAS-Q

**Figure 1:**
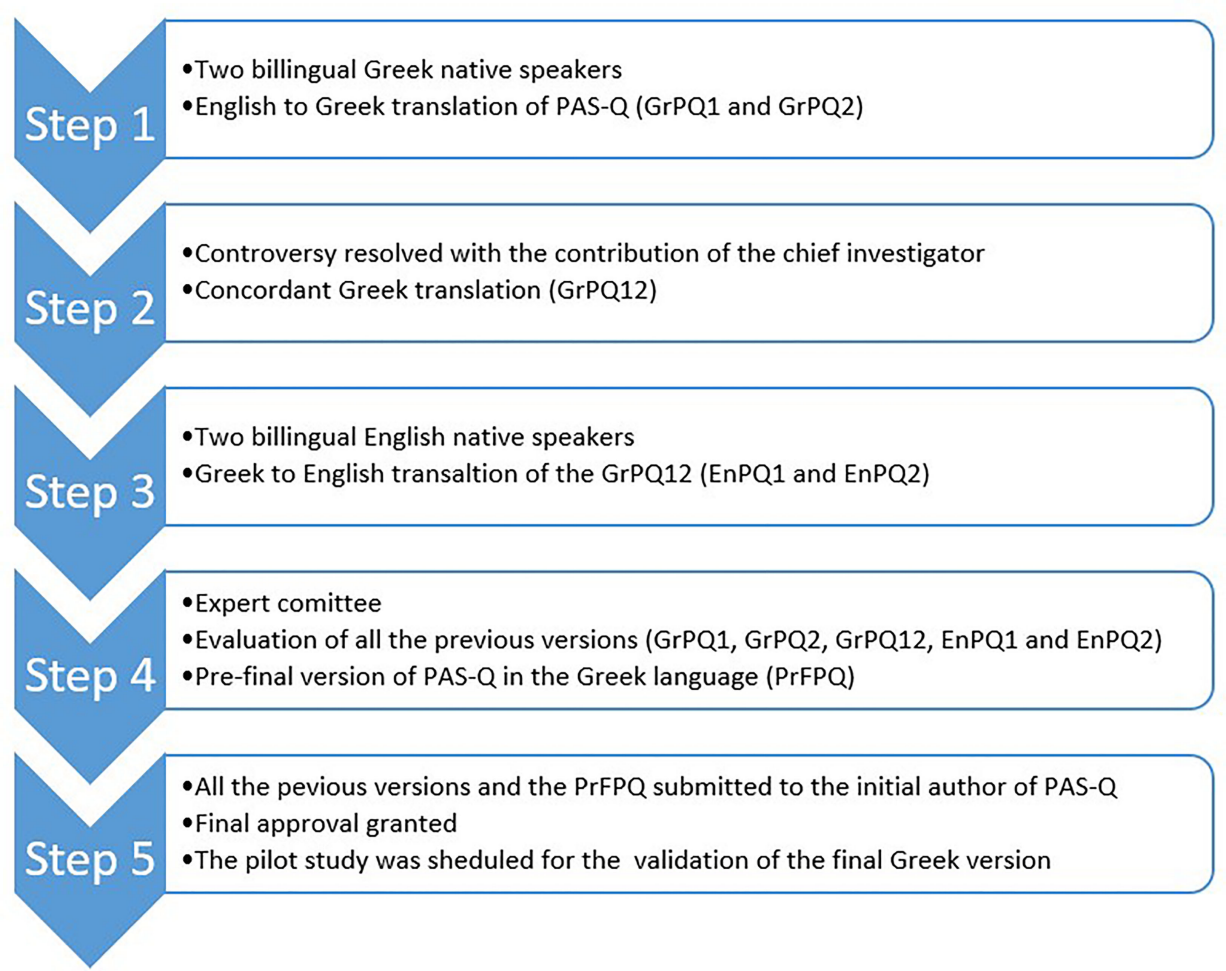
Translation and adaption procedure of PAS-Q.

The authors translated the PAS-Q into the Greek language based on the method introduced by Beaton et al. and following the World Health Organization (WHO) guidelines (3, 4). All the steps followed during translation are presented in **Figure 1**. The first step involved a “forward” translation of the PAS-Q from English into Greek by two independent, bilingual translators who live and work in Greece and whose native language is Greek. The first translator was an experienced endocrinologist and the second one a Medical School post-graduate student whose research domain was the evaluation of the impact of parathyroidectomy on patients with PHPT. These translators were informed of the use and purpose of the questionnaire and specific clinical terms. Each translator created an independent version of PAS-Q in the Greek language - GrPQ1 and GrPQ2, each reporting the degree of difficulty they experienced and any specific issues which arose. The two versions were reconciled during the second step, with the contribution of the chief investigator, and a concordant translation of the questionnaire - GrPQ12 - was finalized.

The next step involved the recruitment of two other bilingual professional translators, both English native speakers, living in Greece, who were asked to translate the questionnaire (GrPQ12) from the Greek into English language. These two English versions were identified as EnPQ1 and EnPQ2 respectively. This enabled the chief investigator to detect differences and similarities to the original Pasieka et al. version. To this end, every item was evaluated, based on the grading system shown in **Table 1**.

**Table 1:** Grading system for the evaluation of the items included in the questionnaire, regarding the conceptual equivalence between the “forward” and “backward” translated versions.

Grade	Item evaluation
A	Items with perfect conceptual equivalence between the “forward” and “backward” translated versions.
B	Items with sufficient conceptual equivalence between the “forward” and “backward” translated versions. However, one or two different words have been used.
C	Items that have the same meaning, but do not ensure any conceptual equivalence between the “forward” and “backward” translated versions.
D	Total disagreements regarding conceptual equivalence between the “forward” and “backward” translation.

In the fourth step, a committee was formed comprising two professional translators, two endocrinologists, two endocrine surgeons, two medical doctors with M.Sc. degrees in the field of research methodology and all four translators from the previous steps. Taking into consideration all the versions of PAS-Q (GrPQ1, GrPQ2, GrPQ12, EnPQ1 and EnPQ2) and the reports of each translator regarding the rationale behind their decisions in the previous stages of the process, every question was assessed taking into account the contextual, idiomatic, semantic and cultural idioms of the Greek and English languages, in accordance with the WHO guidelines (4). And finally, complete consensus on the pre-final version of the questionnaire (PrFPQ) in the Greek language was reached. In the fifth stage, all versions and adaptation processes were submitted to the initial author of PAS-Q for final approval, following which, the final Greek version was formally adopted, and a pilot study was scheduled for its validation (**Table 2**).

**Table 2:** The original and the final translated version of PAS-Q in Greek.

Question	Original PAS-Q in English	Final Greek Version of PAS-Q
1	Pain in the bones	Έχϵτϵ πόνους στα κόκκαλα;
2	Feeling tired easily	Aισθάνϵστϵ ότι κουράζϵστϵ ϵύκολα;
3	Mood swings	Έχϵτϵ αλλαγές στη διάθϵσή σας (κυκλοθυμικές τάσϵις);
4	Feeling “blue” or depressed	Aισθάνϵστϵ στϵναχώρια ή κατάθλιψη;
5	Pain in the abdomen	Έχϵτϵ πόνους στα κοιλιά;
6	Feeling weak	Aισθάνϵστϵ αδύναμος/η;
7	Feeling irritable	Aισθάνϵστϵ οξύθυμος/η;
8	Pain in the joints	Έχϵτϵ πόνους στις αρθρώσϵις;
9	Being forgetful	Ξϵχνάτϵ καθόλου;
10	Difficulty getting out of a chair or car	Έχϵτϵ δυσκολία να σηκωθϵίτϵ από την καρέκλα ή να βγϵίτϵ από το αυτοκίνητο;
11	Headaches	Έχϵτϵ πονοκϵφάλους;
12	Itchy skin	Έχϵτϵ φαγούρα/κνησμό στο δέρμα;
13	Being thirsty	Έχϵτϵ συχνά αίσθημα δίψας;

### 2.3 Adaption of the PAS-Q to the Greek Population

The reliability of the Greek version of PAS-Q was evaluated by examining its internal consistency and test-retest reliability. Internal consistency represents the level of inter-correlation between each item of the questionnaire or congruity in measurement of the same construct. This is evaluated by using coefficient alpha, also known as Crohnbach’s alpha, which ranges from 0 to 1 with a value of 0 representing no internal consistency, while a coefficient as high as 1 reflects perfect internal consistency (5). However, a Cronbach’s alpha coefficient of more than 0.70 indicates adequate internal consistency (6). Test-retest reliability refers to the degree to which an individual’s responses to the questionnaire items remain relatively consistent across repeated administration of the same questionnaire or alternate questionnaire forms. The duration of the period between the first administration of the PAS-Q and the second one was approximately four weeks during which the patient was scheduled for parathyroidectomy.

### 2.4 Sample

The patient cohort came from the endocrinology outpatient clinics of “AHEPA” and “Hippokration” University Hospitals of Thessaloniki, between February 2017 and February 2019. The study protocol was approved by our institution’s review board. The sample size for field testing of the PAS-Q was calculated using the subject-to-item ratio. The rule of thumb recommends that this ratio should be between 3:1 and 20:1. For this study, a ratio of 4:1 was used. As there were 13 items in the PAS-Q, the minimum required sample size was estimated at 52 participants. Taking a 20% loss-of-follow-up and non-eligibility rate into consideration, this study aimed to include 63 participants (7).

All eligible patients were diagnosed with biochemically proven PHPT, based on the combination of hypercalcemia (total serum calcium concentrations >10.5 mg/dl) with elevated or inappropriately normal parathyroid hormone (PTH) concentrations (>6.9 pmol/L), which fulfilled the criteria for surgery (8). All patients, after having signed and dated the written informed consent, filled in the first questionnaire in the outpatient endocrinology office, as a part of the work-up process.

### 2.5 Statistical Analysis

IBM SPSS Statistics software (25^th^ edition) was used for the statistical analysis. Statistical significance was set at p<0.05. The Kolmogorov-Smirnov test was used to assess the normality of data distribution. The results of continuous variables are presented as means ± standard deviation where normality was assumed, or as medians with their respective inter-quartile range (Q25-Q75) when the data were skewed. For categorical variables, counts and percentages are presented. The Independent Student’s T-Test was applied in order to compare the means between two independent samples with normally distributed data, whereas the Mann-Whitney U Test was used to compare differences between two independent groups, when the dependent variable was either ordinal or continuous, but not normally distributed. Cronbach’s alpha coefficient was estimated for the assessment of internal consistency. Test-retest reliability was evaluated determining intraclass correlation coefficients (ICCs) and their 95% confident intervals (CI) based on a single-rating, absolute agreement 2-way mixed-effects model, for every item of the PAS-Q.

## 3 Results

### 3.1 Translation

The 13 questions of the PAS-Q were translated without any major discrepancy. There were only two points of discussion, regarding the “forward” translation, one relating to question number 7 (feeling irritable) and the other question number 13 (being thirsty), for which consensus was achieved at the first meeting between the two initial translators and the chief investigator during Stage 2.

### 3.2 Pilot Study

#### 3.2.1 Sample

One hundred and fifty-six patients were diagnosed with PHPT. Of those, 96 had an asymptomatic disease and were assigned to active surveillance. The remaining 60 patients met the indication for parathyroidectomy based on the 4^th^ International Workshop criteria for surgical management of PHPT and were scheduled for surgery. Ten patients refused to participate in the study. As a result, 50 patients with asymptomatic PHPT, scheduled for parathyroidectomy, were finally enrolled in our pilot study. Their baseline characteristics are presented in **Table 3**.

**Table 3:** Baseline Characteristics (N=50) and biochemical results at the time of the first administration of the questionnaire (Test) and the second one (Retest).

Gender (males/females)	9/41		
Age (years)		64.0 (12.7)
Weight (kg)		68.0 (17.0)
Height (m)		1.61 (0.07)
BMI (kg/m^2^)		26.1 (6.2)
*Time of assessment*	*Test*	*Retest*	*p value*
PTH (pg/mL)	113.0 (67.5)	106.5 (64.5)	0.97
Total serum calcium (mg/dL)	11.50 (0.97)	11.37 (0.92)	0.32
Serum phosphate (mg/dL)	3.1 (0.6)	3.1 (0.8)	0.66
Serum albumin (g/dL)	4.3 (0.3)	4.2 (0.4)	0.27
24-h urine calcium (mg)	178.0 (111.4)	158.9 (168.0)	0.49
25-hydroxyvitamin-D (ng/ml)	15.9 (10.4)	16.1 (9.9)	0.55
PAS-Q total score	375.5 (349.7)	382.0 (356.3)	0.31

All the data are presented as the median and the interquartile range within the brackets

BMI, body mass index; PAS-Q, Pasieka’s parathyroid assessment of symptoms questionnaire; PTH, parathyroid hormone.

#### 3.2.2 Internal Consistency

Cronbach’s alpha was 0.904, indicating a high level of internal consistency.

#### 3.2.3 Test-Retest Reliability

Agreement between test-retest was excellent for every question of the PAS-Q. **Table 4** shows the ICCs with their 95% CI for every question.

**Table 4:** ICC and 95% confident intervals for every question of PAS-Q.

Question Number	ICC	C.I.
1	0.959	0.929-0.977
2	0.948	0.909-0.971
3	0.850	0.736-0.914
4	0.894	0.814-0.940
5	0.968	0.944-0.982
6	0.918	0.855-0.953
7	0.884	0.796-0.934
8	0.946	0.905-0.969
9	0.925	0.865-0.958
10	0.929	0.861-0.962
11	0.946	0.903-0.970
12	0.863	0.758-0.922
13	0.911	0.843-0.949

CI, confident interval; ICC, intraclass correlation coefficient.

## 4 Discussion

In this study, we translated and adapted the PAS-Q, a PHPT specific diagnostic tool for the evaluation of the health-related quality of life, for the Greek population. A pilot study was then conducted and 50 patients with PHPT were assessed by the Greek version of the PAS-Q. Internal consistency was high and a total agreement between the two assessments was achieved.

PHPT is considered to be the 3^rd^ most frequent endocrine pathology, with an estimated prevalence in the general population close to 1% (9). Patients diagnosed with PHPT suffer from a constellation of symptoms, varying from its classic presentation, primarily affecting the kidneys and bones, to more subtle forms in asymptomatic patients with mild hypercalcemia.

The PAS-Q focuses on measuring the QoL in patients with PHPT, since it has been shown that neurological and psychiatric symptoms of hypercalcemia affect many aspects of a patient’s social interactions and mental health. There is a long list of symptoms, of which the most common are anxiety, depression, personality disorders, weakness, irritability, loss of memory, and loss of focus (10).

The morbidity of many diseases nowadays can be reflected in specific and nonspecific questionnaires designed to characterize the impact of the condition in patients’ quality of life, an important patient-centered outcome. However, most of these questionnaires are initially created in English or other languages and they need to be validated before applying in other countries and populations. The PAS-Q is a questionnaire, created in English and validated in Canada, and its reliability and internal consistency had never been evaluated in a Greek population before. However, it is mandatory that every questionnaire used in the Greek language should be translated and adapted to the Greek population (11, 12). PAS-Q is a useful QoL evaluation tool in the hands of physicians caring for PHPT patients and we believe it is important that its use be extended and validated for use in other than English-speaking settings. Therefore, we aimed to translate and adapt the PAS-Q to the Greek language and culture. Cross-cultural adaptation raised no crucial issues or difficulties. Existing guidelines were meticulously followed to avoid inherent language barriers in Greek during the translation (4, 13). In addition, the authors conducted a pilot study to assess the reliability of the questionnaire in a Greek population of PHPT patients.

Fifty patients with confirmed PHPT were recruited to the pilot study and answered the Greek PAS-Q. The majority were women (n=41), reflecting the gender distribution of PHPT. The test-retest reliability was affirmed based on the data selected from the pilot study. Internal consistency, as assessed by Cronbach’s alpha coefficient, was estimated to have a value of 0.904, reflecting excellent internal consistency (14).

Another parameter in need of evaluation during questionnaire translation and adaptation from one language to another is test-retest reliability, which examines how constant the responders remain across repeated administration of the same questionnaire. Moreover, it reflects the deviation in measurements by the same questionnaire on the same sample of responders under the same circumstances (15). Our results revealed good to excellent reliability of PAS-Q, as shown not only from the ICC but also from its CI, to test its variance and where the reported ICC value is statistically significant.

Finally, during this study, we aimed to perform the cross-cultural adaptation of the PAS-Q by assuring that the conceptual equivalence, the semantic concept and the way the questionnaire is administered were the same between the original and the Greek questionnaires (16). Conceptual equivalence refers to the level to which an item in the questionnaire keeps the same meaning between the two cultures and the people from the different countries understand the same when they read it. The semantic concept has to do with the level and the complexity of the language used in the questionnaire, in order to be understandable by the majority of the target population. Attention was also paid not only to translating and adapting the context of the questionnaire, but also to retaining the way that the PAS-Q is administered and explained.

This study has certain strengths. To begin with, it is the first in the direction of translating and adapting the PAS-Q into another language in order to extend its application worldwide. Great care was taken to employ the correct methodology for proper translation and adaption of an original questionnaire to another language, thus ensuring that the PAS-Q would be a reliable tool for assessing QoL in native Greek patients with PHPT. Secondly, the sample was representative of the Greek population, since our pilot study was conducted in two different tertiary hospitals in a city of about 1.5 million inhabitants.

However, some limitations should also be acknowledged. First, the Greek version of the PAS-Q can only be administered to patients with PHPT who are able to read and understand Greek. This may reflect on selection bias because the patients enrolled in our study were Greeks and therefore this version could only be generalizable to Greek-speaking populations. Second, despite its representativeness, the sample size could be considered relatively small, and the results should be replicated in future studies. Nevertheless, our study aimed primarily to evaluate patient understanding of the purpose, content, wording, instructions and general structure of the PAS-Q and a 4:1 ratio was sufficient for this purpose.

## 5 Conclusions

The Greek version of the PAS-Q, an easy, reliable and disease-specific diagnostic tool, is now ready for use by endocrine surgeons or endocrinologists in Greek-speaking populations, aiming to evaluate the QoL of PHPT patients.

## Data Availability Statement

The raw data supporting the conclusions of this article will be made available by the authors, without undue reservation.

## Ethics Statement

The studies involving human participants were reviewed and approved by Ethical Committee of AHEPA University Hospital, Thessaloniki, Greece (29th SesTop/31stCon/21.12.2016). The patients/participants provided their written informed consent to participate in this study.

## Author Contributions

GT was the chief investigator, wrote the manuscript, and collected the data. KK and TP wrote and corrected the manuscript for its scientific basis. AlM and AC collected the data for the study. CM supervised the translation procedure. KT, PA, MM, and DT were engaged in the translation of the questionnaire. FA was one of the bilingual Greek native speaker translators and edited the final the manuscript. AnM was the Director of the Department of Surgery and provided his permission for this study. All authors contributed to the article and approved the submitted version.

## Conflict of Interest

The authors declare that the research was conducted in the absence of any commercial or financial relationships that could be construed as a potential conflict of interest.

## Publisher’s Note

All claims expressed in this article are solely those of the authors and do not necessarily represent those of their affiliated organizations, or those of the publisher, the editors and the reviewers. Any product that may be evaluated in this article, or claim that may be made by its manufacturer, is not guaranteed or endorsed by the publisher.

## References

[1] Silva BC Cusano NE Bilezikian JP. Primary Hyperparathyroidism. Best Pract Res Clin Endocrinol Metab (2018) 32(5):593–607. doi: 10.1016/j.beem.2018.09.004 30449543

[2] Pasieka JL Parsons LL. Prospective Surgical Outcome Study of Relief of Symptoms Following Surgery in Patients With Primary Hyperparathyroidism. World J Surg (1998) 22:513–8; discussion 518-9. doi: 10.1007/s002689900428 9597921

[3] W.H.O. (WHO). WHO Guidelines on Translation. (2016). Available at: http://www.who.int/substance_abuse/research_tools/translation/en/.

[4] Beaton DE Bombardier C Guillemin F Ferraz MB. Guidelines for the Process of Cross-Cultural Adaptation of Self-Report Measures. Spine (2000) 25:3186–91. doi: 10.1097/00007632-200012150-00014 11124735

[5] Cronbach LJ. Coefficient Alpha and the Internal Structure of Tests. Psychometrika (1951) 16:297–334. doi: 10.1007/BF02310555

[6] Nunnaly J. Psychometric Theory. New York: McGraw-Hill (1978).

[7] Hogarty KY Hines CV Kromrey JD Ferron JM Mumford KR. The Quality of Factor Solutions in Exploratory Factor Analysis: The Influence of Sample Size, Communality, and Overdetermination. Educ psychol Measure (2005) 65:202–26. doi: 10.1177/0013164404267287

[8] Bilezikian JP Brandi ML Eastell R Silverberg SJ Udelsman R Marcocci C. Guidelines for the Management of Asymptomatic Primary Hyperparathyroidism: Summary Statement From the Fourth International Workshop. J Clin Endocrinol Metab (2014) 99:3561–9. doi: 10.1210/jc.2014-1413 PMC5393490 25162665

[9] Press DM Siperstein AE Berber E Shin JJ Metzger R Monteiro R. The Prevalence of Undiagnosed and Unrecognized Primary Hyperparathyroidism: A Population-Based Analysis From the Electronic Medical Record. Surgery (2013) 154(6):1232–7; discussion 1237-8. doi: 10.1016/j.surg.2013.06.051 24383100

[10] Benge JF Perrier ND Massman PJ Meyers CA Kayl AE Wefel JS. Cognitive and Affective Sequelae of Primary Hyperparathyroidism and Early Response to Parathyroidectomy. J Int Neuropsychol Soc: JINS (2009) 15:1002–11. doi: 10.1017/S1355617709990695 19807940

[11] Epstein J Santo RM Guillemin F. A Review of Guidelines for Cross-Cultural Adaptation of Questionnaires Could Not Bring Out a Consensus. J Clin Epidemiol (2015) 68:435–41. doi: 10.1016/j.jclinepi.2014.11.021 25698408

[12] Hall DA Zaragoza Domingo S Hamdache LZ Manchaiah V Thammaiah S Evans C. A Good Practice Guide for Translating and Adapting Hearing-Related Questionnaires for Different Languages and Cultures. Int J Audiol (2018) 57:161–75. doi: 10.1080/14992027.2017.1393565 29161914

[13] Thammaiah S Manchaiah V Easwar V Krishna R. Translation and Adaptation of Five English Language Self-Report Health Measures to South Indian Kannada Language. Audiol Res (2016) 6:153. doi: 10.4081/audiores.2016.153 27588165 PMC4988099

[14] Tsang S Royse CF Terkawi AS. Guidelines for Developing, Translating, and Validating a Questionnaire in Perioperative and Pain Medicine. Saudi J Anaesthesia (2017) 11:S80–9. doi: 10.4103/sja.SJA_203_17 PMC5463570 28616007

[15] Koo TK Li MY. A Guideline of Selecting and Reporting Intraclass Correlation Coefficients for Reliability Research. J Chiropractic Med (2016) 15:155–63. doi: 10.1016/j.jcm.2016.02.012 PMC4913118 27330520

[16] Acquadro C Conway K Hareendran A Aaronson N European Regulatory Issues and Quality of Life Assessment (ERIQA) Group. Literature Review of Methods to Translate Health-Related Quality of Life Questionnaires for Use in Multinational Clinical Trials. Value Health (2008) 11:509–21. doi: 10.1111/j.1524-4733.2007.00292.x 18179659

